# Hepatic Macrosteatosis Is Partially Converted to Microsteatosis by Melatonin Supplementation in *ob/ob* Mice Non-Alcoholic Fatty Liver Disease

**DOI:** 10.1371/journal.pone.0148115

**Published:** 2016-01-29

**Authors:** Alessandra Stacchiotti, Gaia Favero, Antonio Lavazza, Igor Golic, Marija Aleksic, Aleksandra Korac, Luigi Fabrizio Rodella, Rita Rezzani

**Affiliations:** 1 Anatomy and Physiopathology Division, Department of Clinical and Experimental Sciences, University of Brescia, Viale Europa 11, 25123, Brescia, Italy; 2 Interdipartimental University Center of Research “Adaption and Regeneration of Tissues and Organs- (ARTO)”, University of Brescia, Italy; 3 Istituto Zooprofilattico Sperimentale della Lombardia e dell’Emilia Romagna, Via A. Bianchi 7/9, 25124, Brescia, Italy; 4 Center for Electron Microscopy, Faculty of Biology, University of Belgrade, Belgrade, Republic of Serbia; Bambino Gesù Children's Hospital, ITALY

## Abstract

**Background:**

Obesity is a common risk factor for non-alcoholic fatty liver disease (NAFLD). Currently, there are no specific treatments against NAFLD. Thus, examining any molecule with potential benefits against this condition emerged melatonin as a molecule that influences metabolic dysfunctions. The aim of this study was to determine whether melatonin would function against NAFDL, studying morphological, ultrastuctural and metabolic markers that characterize the liver of *ob/ob* mice.

**Methods:**

Lean and *ob/ob* mice were supplemented with melatonin in the drinking water for 8 weeks. Histology and stereology were performed to assess hepatic steatosis and glycogen deposition. Ultrastructural features of mitochondria, endoplasmic reticulum (ER) and their juxtapositions were evaluated in livers of all experimental groups. Furthermore, hepatic distribution and expression of markers of ER and mitochondria (calnexin, ATP sintase β, GRP78 and CHOP) and metabolic dysfunction (RPB4, β-catenin) and cellular longevity (SIRT1) were analyzed.

**Results:**

Melatonin significantly reduced glycemia, identified also by a decrease of hepatic RBP4 expression, reversed macrosteatosis in microsteatosis at the hepatic pericentral zone, enlarged ER-mitochondrial distance and ameliorated the morphology and organization of these organelles in *ob/ob* mouse liver. Furthermore, in *ob/ob* mice, calnexin and ATP synthase β were partially restored, GRP78 and CHOP decreased in periportal and midzonal hepatocytes and β-catenin expression was, in part, restored in peripheral membranes of hepatocytes. Melatonin supplementation to *ob/ob* mice improves hepatic morphological, ultrastructural and metabolic damage that occurs as a result of NAFLD.

**Conclusions:**

Melatonin may be a potential adjuvant treatment to limit NAFLD and its progression into irreversible complications.

## Introduction

Obesity is a worldwide epidemic and the fifth leading cause of death [1]. Obesity negatively impacts the function of many organs including cardiovascular and metabolic systems [2]. Moreover, obesity is a common well-documented risk factor for non-alcoholic fatty liver disease (NAFLD). This latter condition is a chronic liver disease, which includes a spectrum of hepatic pathologies and can involve cirrhosis. Moreover, NAFLD is characterized by macrovescicular steatosis and hepatocyte ballooning. These alterations are similar to those observed in alcoholic liver disease in humans even if they occur in the absence of alcohol consumption [3]. The liver is mainly involved in NAFLD because it functions in glycogen deposition, fatty acid oxidation, cholesterol transport and modulation of bile flux [4,5]. In particular, the endoplasmic reticulum (ER) of hepatocytes plays an important part in fatty acid synthesis and cholesterol metabolism. The relationship between ER stress and fatty liver is a bilateral one: steatosis has been shown to promote ER stress and, conversely, the ER stress response leads to steatosis. Current evidence strongly supports an important role for ER stress in NAFLD [6].

Globally, NAFLD affects numerous people around the world; its frequency is increasing possibly due to changes in dietary habits and the common prevalence of sedentary lifestyles [7,8]. Current, NAFLD treatments involve correcting diet, exercise and drugs, including metformin, statins and fibrates. However, these drugs have a variety of adverse effects or contraindications and there is still no consensus on the most effective drug therapy for NAFLD [9]. Therefore, new candidates, such as active endogenous molecules with high efficiency and minimal side effects are urgently needed for the treatment of NAFLD.

Retinol binding protein 4 (RBP4) is produced in different tissues, including adipose tissue, liver and muscles. Mansouri et al. [10] reported that increased serum level of RBP4 in diabetic rats might be a consequence of its elevated mRNA and protein expression in the liver and adipose tissue, with the highest expression level in the liver followed by adipose tissue. Moreover, circulating RBP4 is elevated in obese subjects compared to lean individuals. Additionally, RBP4 mRNA in visceral and subcutaneous abdominal adipose tissue is higher in obese patients compared to lean subjects [11].

Calnexin (CNX) is a 90-kDa type I integral ER transmembrane chaperone that transiently binds newly-synthesized monoglucosylated and misfolded glycoproteins, promoting their folding and oligomerization [12,13]. ATP-synthase subunit β (ATPSβ) is a novel biomarker of mitochondrial dysfunction. More recently, it has been demonstrated its role in regulation of hepatic ATP content and glucose metabolism in diabetic mice. ATPSβ expression and ATP content were both reduced in the liver of type 1 and type 2 diabetic mice [14].

G protein-coupled receptor (GRP78), the master resident ER chaperone, and its downstream target CCAAT/enhancer binding protein-α (C/EBP) homologous protein (CHOP) are involved in the development of NAFLD in obese animal models and patients [15,16]. In particular, the mitochondria associated membranes area (MAM), a subdomain of the ER, is crucial for lipid transfer, regulation of mitochondrial shape and modulation of ER stress in metabolic human diseases [17].

Sirtuins (SIRTs) are enzymes with NAD^+^-dependent class III histone deacetylase activity that regulate longevity and metabolism in mammalians [18–20]. SIRT1 limits obesity by enhancing fatty acid oxidation and mitochondria biogenesis due to interaction with proliferators-activated receptor coactivator-1α (PGC-1α) [21].

Beta-catenin (β-catenin), an indicator of hepatic metabolism is involved in regeneration, resistance to hypoxia, apoptosis, steatosis, modulation of zonation and cholesterol metabolism [22,23]. Consequently, β-catenin signaling is essential for an optimum hepatic homeostasis. Further, it has been demonstrated that its dysregulation is evident in aberrant hepatic growth during various liver tumors [24].

Melatonin, an indoleamine derived from L-tryptophan and secreted from pineal gland, shows a wide range of physiological and pharmacological functions including beneficial effects in metabolic diseases and their cardiovascular complications [25–28]. Exogenous melatonin supplementation is safe and, due to its amphipathic nature, easily penetrates into every subcellular site. Furthermore, melatonin maintains biological membrane fluidity and it acts as an antioxidant and reactive oxygen species (ROS) scavenger also at the mitochondrial level [29]. Moreover, it counteracts adipogenesis by stimulating thermogenesis, insulin sensitivity, glucose uptake and ameliorating liver functions in different metabolic and physiological conditions [30]. Recent studies reveal that melatonin directly regulates metabolic parameters in experimental and clinical studies [30–33], restores sleep rhythm in obese patients [34] and the hormonal flux in high fat diet rodents [35]. Nevertheless, melatonin effects are linked to circadian rhythm, dose, administration route and the presence of specific receptors [36]. Indeed, melatonin improves hepatosteatosis, but does not reduce liver weight when intra-peritoneally administered in *ob/ob* mice at 18 weeks of age [37]. Although, numerous papers showing that melatonin is associated with reduction of adiposity and body mass [30], its roles and its action mechanisms are not fully understood. Thus, the aim of this study was to determine how melatonin might act as a potential therapeutic tool for this health problem. For this purpose, we used *ob/ob* mice, which have a defect in leptin synthesis and reproduces many of the metabolic disturbances present in NAFLD patients [38] which allowed us to investigate the effects of melatonin administration on liver morphology and function. Herein, we considered and evaluated a variety of markers listed above involved in metabolic functions, oxidative stress and cellular longevity of hepatocytes. Our results indicate that melatonin administration to *ob/ob* mice improves hepatic morphological and metabolic damages determined by NAFLD that are rising because of an increasing prevalence of obesity and metabolic syndrome [39].

## Materials and Methods

### Experimental design

The experiments were conducted according the National Ministerial guidelines, minimizing any sufferance. In particular, the experimental animal project was approved by the Committee for the Care and Use of Animals of the University of Brescia, Italy and by the Italian Ministry of Health. 40 male mice at 4 weeks of age grouped included 20 lean (B6.V-(lean)/Ola Hsd) and 20 obese leptin-deficient animals (B6.V-Lep^ob^/OlaHsd), purchased from Harlan Laboratories S.r.l., Udine, Italy, were studied. Animals were housed in an approved facility with 12h dark/12h light and constant temperature with a standard rodent diet provided *ad libitum*. The animals received, for 8 weeks from 5^th^ to 13^th^ week, of life supplemented vehicle or melatonin dissolved in drinking water at the final dose of 100 mg/kg/day. These dose and duration of melatonin treatment had previously been shown to improves glucose homeostasis by restoring the vascular action of insulin in a study of Sartori et al.[40]. Melatonin was dissolved in 1% ethanol and diluted in drinking water. Fresh melatonin and vehicle solutions were prepared twice a week and the melatonin content was adjusted to the body weight of individual animals throughout the study period. Body weight, water consumption and blood glucose were regularly monitored, as describe in detail in the following paragraph. At the end of the treatment, mice were sacrificed by cervical dislocation and liver was removed and fixed in buffered formalin for 24 hours and embedded in paraffin wax for histological and immunohistochemical stainings or fixed in 2.5% glutaraldehyde in 0.1M cacodilate buffer, post-fixed in osmium tetroxide and embedded in Epon resin for ultrastructural analysis or properly homogenized and then processed for the enzyme-linked immunosorbent assay (ELISA).

More details on chow composition, food intake and metabolic parameters have been reported in our previous studies [25,31,41].

### Body weight and blood glucose evaluation

Body weight was measured twice a week, before melatonin replace, always at the same time of day and body weight gain was calculated as the difference between body weight measured at the beginning and at the end of all the period of treatment. Furthermore, blood glucose was collected via tail-snip and measured using a glucose test strip reader.

### Histological analyses

Paraffin sections (7μm thick) cut with a microtome were collected on poly-D-lysine-coated slides, deparaffinized, rehydrated and stained using haematoxylin or PAS for histopathological analysis. Steatosis was assessed on hematoxylin-eosin stained sections by counting randomly chosen 200 lipid droplets/groups and selecting their diameter (μm), area (μm^2^) and volume density (%) at a final magnification of 400X in pericentral hepatocytes. PAS positive glycogen and glycoproteins inside hepatocytes was estimated according to hepatic zonation at final magnification of 200X and expressed as arbitrary units (AU). The morphometrical analyses were performed by two different observers blinded to the experimental group, using a computer image analysis software (Image J-Image Processing and Analysis in Java).

### Immunohistochemical analysis

Paraffin sections of liver were deparaffinized, rehydrated and subjected to antigen retrieval in 0.01M sodium citrate buffer (pH 6.0) in a microwave oven and then the sections were treated in 3% hydrogen peroxide to block endogenous peroxidase activity. The sections were incubated then with normal serum from species producing the secondary antibody and subsequently with primary polyclonal antibodies against SIRT1, GRP78 and CHOP (Santa Cruz Biotechnology, Santa Cruz, CA, USA), diluted 1:150, 1:300 and 1:50 respectively and monoclonal antibodies against β-catenin (BD Transduction, San Jose, CA, USA) and RBP4 (Abcam, Cambridge, UK), diluted 1:50 and 1:100 respectively. After washing in tris buffered saline (TBS), specific secondary antibodies were applied and then incubated in avidin-biotin-peroxidase complex according to manufacturer instructions (ABC-peroxidase kit Vector Labs, Burlingame, CA, USA). Finally, the sections were incubated in a solution of 0.05% 3–3’-diaminobenzidine tetrahydrochloride (Sigma Aldrich, St. Louis, MO, USA) and 0.33% hydrogen peroxide [42,43]. All sections were finally counterstained with haematoxylin, dehydrated and mounted. Negative controls were performed by omitting the primary antibody step.

Immunostaining intensity, expressed as arbitrary units (AU) or percentage of immunopositive nuclei, was evaluated by two observers blinded to the specific experimental group. 20 random fields with the same area were estimated using an image analyzer (Image Pro Plus, Milan, Italy).

### Sirtuin1 ELISA assay

Liver contents of SIRT1 was measured using a commercial mouse ELISA kit (AbCam, Cambridge, UK), according to manufacturer’s guidelines. In particular, the optical density was determined at 450 nm in a microplate reader and the SIRT1 levels were expressed as ng/ml.

### Immunofluorescence analysis

Deparaffinized and rehydrated sections were incubated in citrate buffer for 10 minutes in a microwave oven to reveal antigens. After washings in TBS, sections were incubated with 10% normal goat serum (Abcam, Cambridge, UK) with 1% bovine serum albumine for an hour at room temperature. This was followed by an overnight incubation at 4°C within the following primary antibodies: calnexin and ATPSβ (both diluted 1:100; Abcam, Cambridge, UK). After washings in TBS with 0.1% Tween-20 (TBS-T), sections were labeled with appropriate fluorochrome-conjugated secondary antibody mixture. Calnexin was labeled with Alexa Fluor 488 secondary antibody (diluted 1:400; Life Technologies, Carlsbad, CA, USA) and ATPSβ was labeled with Alexa Fluor 633 antibody (diluted 1:400; Life Technologies, Carlsbad, CA, USA). After, the slides were counterstained with the nuclear stain propidium iodide (diluted 1:1000; Sigma Aldrich, Seelze, Germany) for 5 minutes. As a final step, slides were washed in TBS and mounted with Mowiol (Sigma Aldrich, Seelze, Germany). Confocal images were acquired with Leica TCS SP5 II microscope (Leica Microsystems, Wetzlar, Germany) in sequential mode to avoid crosstalk between channels. The double stained sections were excited with 488 and 633 nm lasers, respectively. Nuclei were visualized using 543 nm laser and blue false-colored for clear distinction of green/red channels. Negative controls were performed by the omission of primary antibodies. Immunofluorescence intensity, expressed as percentage of immunopositivity normalized to lean value, was determined using a LAS AF software (Leica Microsystems, Wetzlar, Germany), by two observers blinded to the specific experimental group.

### Transmission electron ultrastructural analysis

Semithin (1μm-thick) sections cut with an ultramicrotome (Ultacut E- Leica, Microsystems S.r.l., Milan, Italy) usung a diamond glass were collected, stained by toluidine blue and examined by light microscopy at 1000x using an immersion oil-objective to detect macrosteatosis and microsteatosis (Liquori et al., 2009). For ultrastructural analysis, thin (80 nm-thick) sections obtained from periportal zone 1 and pericentral zone 3 were collected on 200 meshes-formvar coated copper grids, double stained with uranyl acetate and lead citrate and photographed using FEI Tecnai G2 Spirit or CM12 TEM transmission electron microscopes (FEI Instrumentation Company, Eindhoven, The Netherlands) at 80kV. Evaluation of ER-mitochondria distance and mitochondria distribution was performed at pericentral zone 3 level [44].

Mitochondrial diameter was calculated, by two blinded observers, as the average diameter per mitochondrion and 250 mitochondria per group were randomly selected. All above data were measured on digital photomicrographs at final magnification of 15.000X with the aid of an image analysis software (Image J- Image Processing and Analysis in Java). Moreover, fifty ER-mitochondria distance were randomly chosen for each group by two blinded investigators unaware of the experimental group. Further, mitochondria-ER distance were observed at 135.000X by a “Veleta” camera (Olympus Soft Imaging Solutions, Munster, Germany) and measured by an Olympus Soft Imaging System (iTEM FEI 5.1.30), by two different observers blinded to the specific experimental group.

### Statistical evaluation

Data were pooled as mean values ± standard errors. The ANOVA corrected by Bonferroni test was used to determine differences between groups comparing the internal variability of group with the variability among all experimental groups. A *p*<0.05 was considered statistically significant. All assays were performed in triplicate, data were randomly collected and the analyses were performed using OriginPro 9.1 software.

## Results

In this study, *ob/ob* mice treated with melatonin were compared with *ob/ob* animals and with lean mice treated or not treated with melatonin. In our previous reports [25,31,41], we published the food and weekly water intake values of these experimental groups. Herein, we report only metabolic parameters, including body weight, mass gain, total water consumption and blood glucose concentration, these data indicate the general health condition of also melatonin treated experimental groups and underline that there are no side effect of melatonin supplementation. In detail, body weights were significantly higher in *ob/ob* compared to that in control animals. Moreover, we observed a reduction of body weight in *ob/ob* mice treated with melatonin, although it was not significant. These observations were confirmed also by the body weight gain observed from the 5^th^ to 13^th^ week of life (8 weeks of treatment), that was significantly higher in *ob/ob* mice respect lean mice treated and not treated with melatonin and decreased a bit (not significantly) after melatonin supplementation. Furthermore, *ob/ob* mice had a total water consumption significantly higher respect lean mice and melatonin administration decreased significantly water consumption in obese treated mice. In contrast lean mice treated with melatonin did not modify water intake throughout the treatment period, as confirmed also by water weekly consumption showed in our previous study [25]. Melatonin supplementation in obese mice also reduced significantly blood glucose to about 300–350 mg/dl *vs* 400 mg/dl of the hyperglycemic *ob/ob* mice (*p*<0.05). Glycemic value was significantly higher with respect control mice treated and not treated with melatonin, which had glucose concentrations less than 200 mg/dl. All these reported above data are summarized in Tables 1 and 2. Furthermore, lean animals treated or not treated with melatonin had similar both weight and glucose levels, so we refer to both animal groups as “control”.

**Table 1:** Body weight and mass gain of each experimental groups.

	Body weight (g)			Body weight gain (%)
	*5 ws*	*9 ws*	*13 ws*	*5–13 ws*
**Lean**	23.8±0.63	28.8±0.16	30.8±0.16	22.73
**Lean + MEL**	23.9±0.27	27.8±0.36	31±0.27	22.91
***ob/ob***	24.8±0.26	48.3±0.31 * +	54.2±0.09 * +	54.24 * +
***ob/ob* + MEL**	24.1±0.29	46.2±0.27 * +	51.9±0.25 * +	53.57 * +

The values are expressed as mean values ± standard errors.

* *p*<0.05 vs lean mice

^+^ *p*<0.05 vs lean mice treated with melatonin.

**Table 2:** Water consumption and blood glucose concentration of each experimental groups.

	Total water consumption (ml/8 ws)	Glucose concentration (mg/dl)		
	*5–13 ws*	*5 ws*	*9 ws*	*13 ws*
**Lean**	218.8±0.38	178±2.8	176±1.89	187±2.93
**Lean + MEL**	221.76±0.32	178±3.9	179±2.46	185±4.09
***ob/ob***	512.16±0.21 * +	255±3.22 * +	376±1.86 * +	391±1.61 * +
***ob/ob* + MEL**	425.92±0.36 * +#	252±3.2 * +	307±1.48 * + #	341±3.11 * +#

The values are expressed as mean values ± standard errors.

* *p*<0.05 vs lean mice

^+^ *p*<0.05 vs lean mice treated with melatonin

^#^ *p*<0.05 vs *ob/ob* mice.

In the hepatic parenchyma, metabolic functions are located heterogeneously in the periportal (comprising hepatocytes surrounding the distal branches of the portal vein) and pericentral (comprising hepatocytes surrounding the proximal branches of the central vein) zones of the liver lobule [45]. Liver zonation is fundamental for proper functioning of this organ [46]. In rodents and humans three zones of equal width, extending from the portal area (Glisson’s sheath) to the central vein, are distinguished: zone 1-periportal, zone 2-intermediate, zone 3-pericentral [47]. The lobular pattern, with the central vein in the centre and the portal triads in the periphery, was clearly evident in all experimental groups. The control mice showed a radially arranged rays of hepatocytes, regularly directed from the central vein of each lobule towards its periphery. In particular, the morphological analysis of the liver showed significantly fat droplets deposition in *ob*/*ob* mice respect control group, clearly localized in the pericentral area (zone 3) of liver lobules, resulting in enlarged fat-laden hepatocytes, evaluated in detail in this study. The same hepatic zone was greatly associated with steatosis also in adult patients with NAFLD [48].

**Fig 1:**
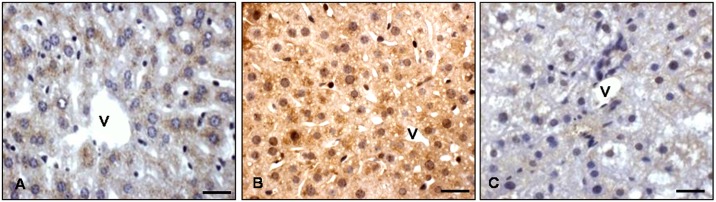
Liver RBP4 immunostaining. Hepatic immunostaining for RPB4 in lean (A), *ob/ob* (B) and *ob/ob* treated with melatonin (C) mice. Scale bars: 20 μm. (v) identifies hepatic centrolobular vein.

To better evaluate the role of glycemic value in *ob/ob* mice, we studied also RBP4 expression which is linked to blood glucose concentration [10]. Interestingly, RBP4 immunostaining was mainly located in pericentral zone 3 of the lean liver and it was essentially undetectable in other lobular zones of control mice (Fig 1A). In contrast, in *ob/ob* mice showed an extensive, significative and strong RBP4 signal diffusely distributed in every lobule from periportal zone 1 to pericentral zone 3 both at the nuclear and cytoplasmic level (Fig 1B). Remarkably, after melatonin supplementation in *ob/ob* mice, RBP4-immunoreaction was significatively restricted to pericentral hepatocytes, mainly in the cytoplasm where the staining was weak (Fig 1C).

**Fig 2:**
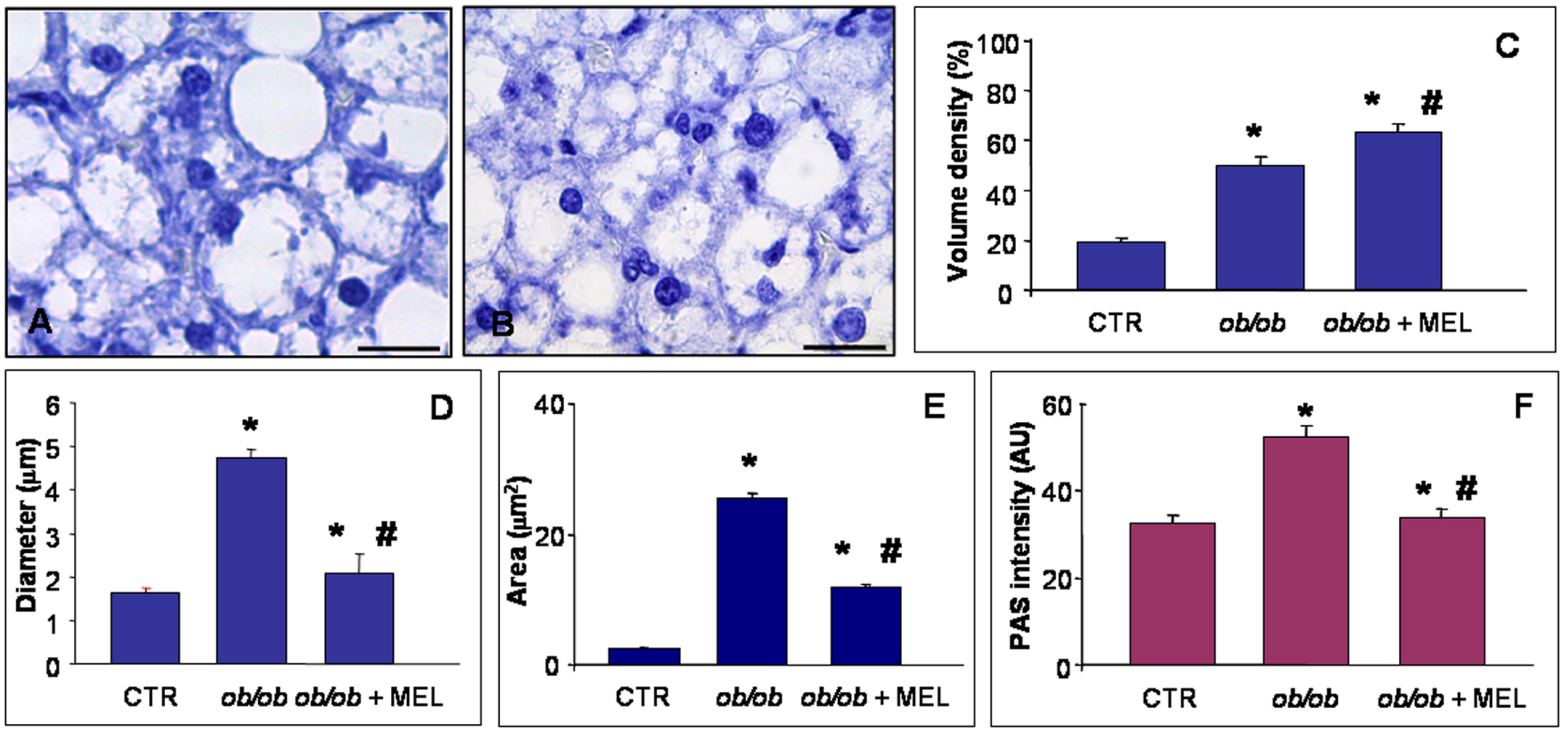
Liver lipid droplets and glycogen. Hepatic histological staining of *ob/ob* (A) and *ob/ob* treated with melatonin (B), showing macrosteatosis and microsteatosis respectively. Haematoxylin staining. Scale bars: 20 μm. The graphs summarized the morphometrical analyses of lipid droplet volume density (C), diameter (D) and area (E) and PAS glycogen intensity (F). * *p*<0.05 vs lean mice and # *p*<0.05 vs *ob/ob* mice.

**Fig 3:**
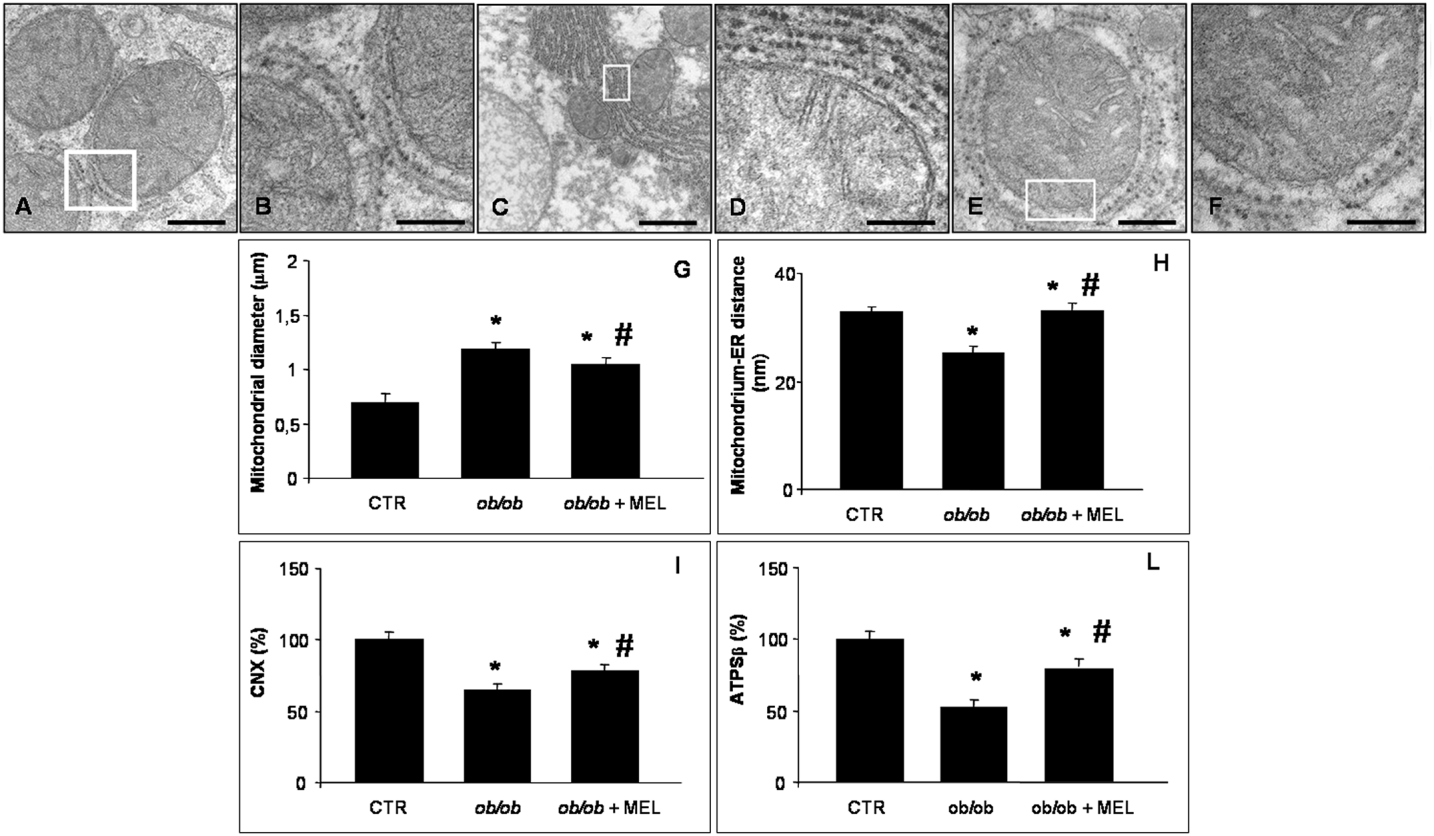
Hepatic mitochondria and endoplasmic reticulum. Transmission electron microscopy photomicrographs of lean (A, B), *ob/ob* (C, D) and *ob/ob* treated with melatonin mice (E, F) showed hepatic mitochondria and endoplasmic reticulum in all experimental groups. The white boxes identify the mitochondria-endoplasmic reticulum contact. Scale bars: 500 nm for A and E; 200 nm for B, D and F and 1 μm for C. The graphs summarized the morphometrical analyses of mitochondrial diameter (G), mitochondrium-endoplasmic reticulum distance (H) and of immunostaining evaluation for calnexin (I) and ATP synthase β (L). * *p*<0.05 vs lean mice and # *p*<0.05 vs *ob/ob* mice.

After observing that treatment of *ob/ob* mice with melatonin resulted in a reduction of the parameters listed above, we investigated morphological and ultrastructural features of the liver. Haematoxylin staining of the liver in control mice showed normal cytoarchitecture of the parenchyma and periportal hepatocytes with occasional steatosis (less than 10%-grade 1). In contrast, in *ob/ob* mice, significative hepatocyte ballooning and extensive steatosis was evident in every lobular zone, but mainly in the midzonal area where it appeared as a mixture of macrosteatosis and microsteatosis (Fig 2A). When quantified, in obese liver more than 85% of hepatocytes were filled with lipids (steatosis grade 3). By contrast, in *ob/ob* mice treated with melatonin, macrosteatosis shifted to microsteatosis with 45% of the hepatocytes exhibiting steatosis (grade 2), so with a significant decrease of macrosteatosis (Fig 2B). This trend was confirmed by detailed quantitative analysis of hepatosteatosis expressed as lipid droplet volume density, diameter and area (Fig 2C–2E). Moreover, the measurement of glycogen deposition, shown in Fig 3F, was moderate in periportal and pericentral hepatocytes of control mice, intense in *ob/ob* mice and significantly attenuated by melatonin supplementation.

Transmission electron microscopic observations revealed “normal” hepatocytes and regular-shaped organelles in the control mice. In particular, the mitochondria appeared with regular cristae encircled by short ER cisternae (Fig 3A and 3B). On the contrary, the *ob/ob* hepatocytes were largely filled with lipid droplets, so mitochondria were restricted in a narrow rim beneath the plasma membrane. Lipid droplets of different sizes sometimes presented a dense osmophilic content. Mitochondria were heterogeneous in size and shape, with a greater diameter respect controls and they were often seen as clusters with a dense matrix devoid of inclusions and with short cristae. In *ob/ob* mice the elongated rough ER (RER) cisternae were closely associated with mitochondria and a significantly reduce distance between ER and mitochondria was observed respect the control mice, underlining an abnormal increase in MAM formation (Fig 3C and 3D). In *ob/ob* mice treated with melatonin, hepatocytes in pericentral and midzonal area contained significantly fewer lipid droplets, so mitochondria occurred also in the perinuclear area. In particular, in the pericentral zone 3 round mitochondria with short peripheral cristae and elongated types with a reduced diameter and an irregular profile were observed. RER was well organized often in short and regular cisternae and enlarged, compared with *ob/ob* mice not melatonin treated, and the mitochondria were less frequently attached to ER respect mitochondria collected from obese mice (Fig 3E and 3F). Quantitative analysis of the mitochondrial diameter (Fig 3G) and of the distance between ER and mitochondria (Fig 3H), an indirect marker of MAM, taken together with ultrastructural evaluation indicate that obesity leads to significant alterations in hepatic mitochondria and ER structure and enhance MAM formation, as confirmed also by Arruda et al. [55].

**Fig 4:**
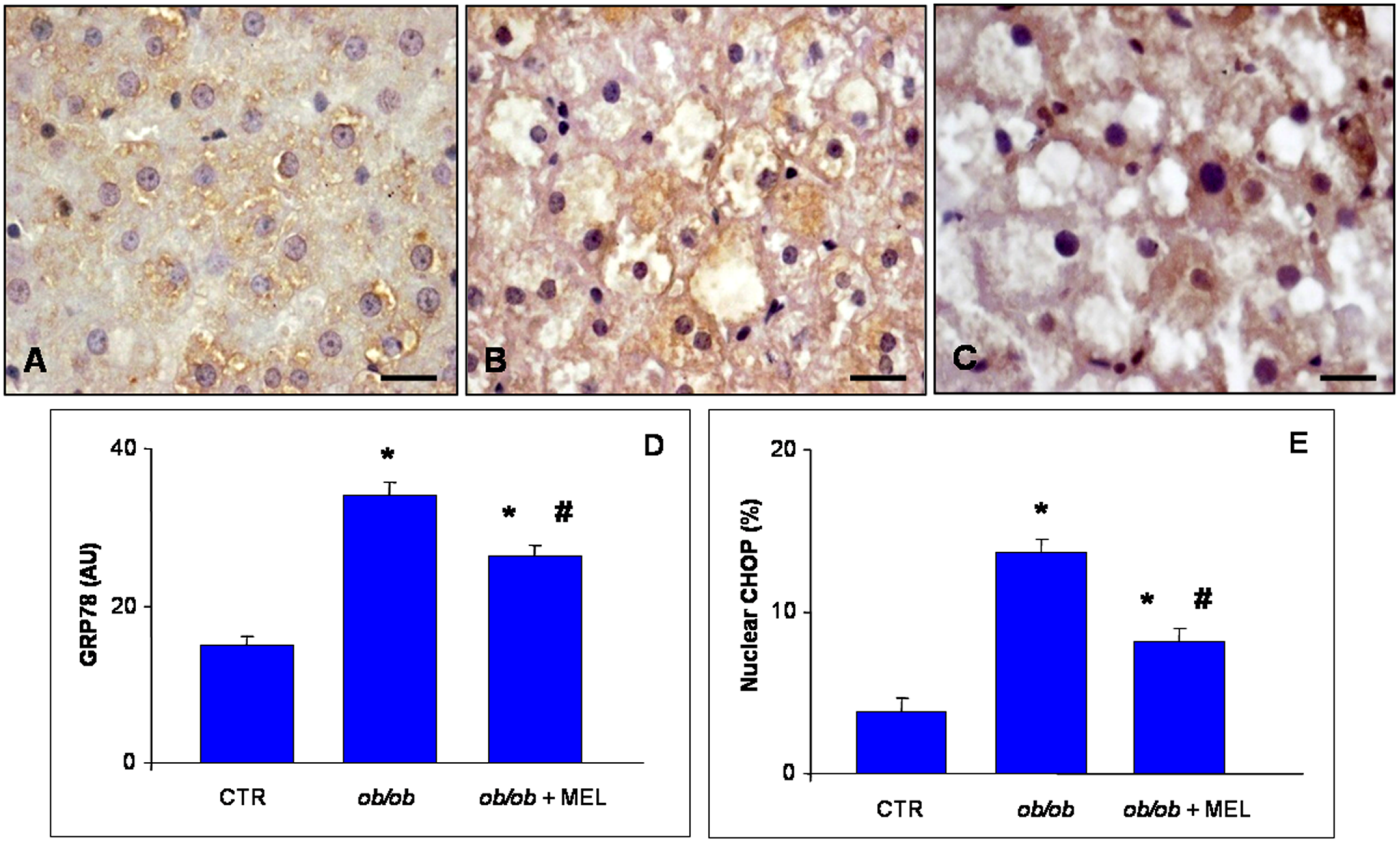
Liver GRP78 and CHOP immunostainings. Hepatic immunostaining for GRP78 in lean (A), *ob/ob* (B) and *ob/ob* treated with melatonin (C) mice. Scale bars: 20 μm. (v) identifies hepatic centrolobular vein. The graphs summarized the immunostaining evaluation for GRP78 (D) and nuclear CHOP (E). * *p*<0.05 vs lean mice and # *p*<0.05 vs *ob/ob* mice.

To confirm whether ER and mitochondria were involved in alterations of hepatic parenchyma, immunofluorescence analysis of CNX, ATPSβ, GPR78 and CHOP was performed. In detail, we demonstrated an intense and significant positive staining of CNX and ATPSβ in control mice, that was significantly attenuated in *ob/ob* mice and partly, but meaningful recovered by melatonin. These results were summarized in Fig 3I and 3L, respectively. Moreover, we analyzed the distribution in hepatocytes of specific ER stress markers: GRP78 and CHOP. Especially in zone 2, GRP78 cytoplasmic staining intensity was moderate in control mice (Fig 4A), almost doubled, so significantly express, in *ob/ob* mice (Fig 4B) and significantly decreased after melatonin supplementation (Fig 4C); as confirmed and then summarized in Fig 4D. No nuclear positivity was observed for GRP78, whereas we observed a moderate nuclear CHOP immunostaining in control mice, strong, diffuse and significant in mid-zonal hepatocytes of *ob/ob* mice and weak and limited in *ob/ob* plus melatonin mice, so significantly reduced respect obese mice not treated. The percentage of CHOP positive nuclei was less than 4% in control mice, enhanced to 15% in *ob/ob* mice and decreased to 7% after melatonin intake, thus showing significant difference between *ob/ob* mice treated and not with melatonin respect controls and between obese mice treated with melatonin compared with untreated *ob/ob* animals (Fig 4E).

**Fig 5:**
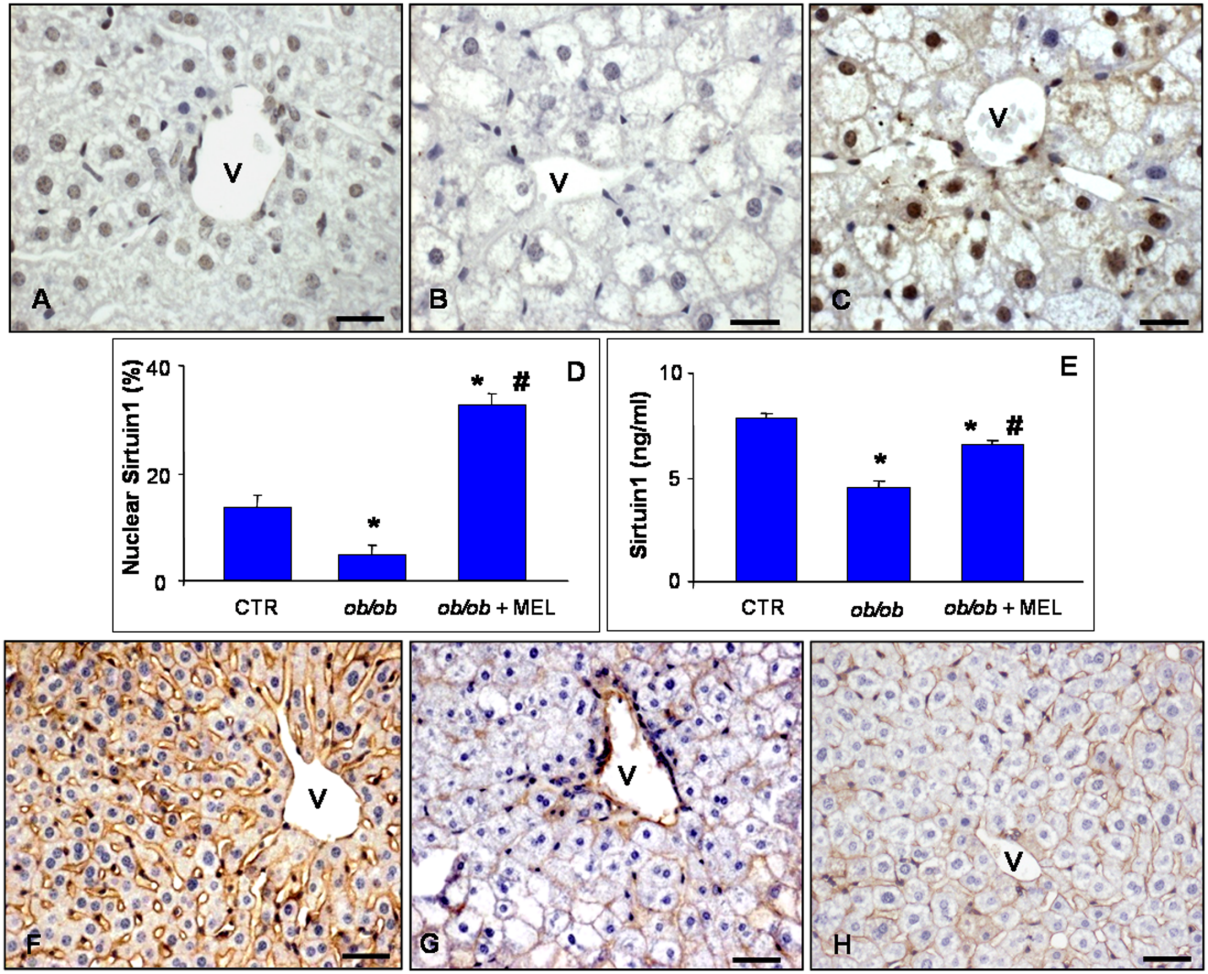
Liver SIRT1 and β-catenin immunostainings. Hepatic immunostaining for sirtuin 1 (A-C) and β-catenin (F-H) in lean (A, F), *ob/ob* (B, G) and *ob/ob* treated with melatonin (C, H) mice. Scale bars: 20 μm. (v) identifies hepatic centrolobular vein. The graphs summarized the sirtuin 1 immunostaining evaluation (D) and quantitative protein concentration in liver homogenate (E). * *p*<0.05 vs lean mice and # *p*<0.05 vs *ob/ob* mice.

Based on these observations and to best correlate the morphological changes, the protective role of melatonin on hepatic parenchyma alterations and the related underlying mechanisms, we analyzed different markers involved in cellular longevity and hepatic metabolism: SIRT1 and β-catenin. Regarding SIRT 1 expression, we demonstrated that SIRT1 was detected in nuclei of lobular zone 3 and zone 1 axis at a moderate level in control mice (Fig 5A). In contrast, in obese mice, SIRT1 immunoreaction was significantly barely detectable in the nuclei of hepatocytes of the same areas (Fig 5B). After melatonin supplementation an evident and meaningful signal was restricted to the nuclei of in pericentral zone 3 hepatocytes (Fig 5C). This data also were confirmed by quantitative evaluation of SIRT1 nuclear immunoassaying (Fig 5D) and, importantly, also by the quantitative measurement of SIRT1 protein level in tissue homogenate (Fig 5E).

To better evaluate metabolic changes in the liver of *ob/ob* mice, we studied also β-catenin. In control mice, β-catenin signal was moderately to strongly expressed at the boundary of hepatocytes in the pericentral zone 3 (Fig 5F). In contrast, in *ob/ob* mice, β-catenin immunoreaction was significantly weak and sometimes barely detectable (Fig 5G). However, in *ob/ob* mice treated with melatonin, β-catenin immunopositivity was partially, but significantly, recovered in pericentral zone 3 hepatocytes showing a moderate expression (Fig 5H).

## Discussion

The results show that melatonin effectively reduces hepatic damage caused by NAFLD as related to obesity and metabolic syndrome. These improvements were evaluated at morphological and metabolic levels. In particular, we demonstrated that melatonin reduced body weight, blood glucose and macrosteatosis in *ob/ob* mice. In particular, in fatty liver of *ob/ob* mice, lipid droplets are large and filled with triglycerides, reducing fatty acid oxidation and alterating microcirculation as reported by El-Badry et al. [49]. As already documented, melatonin plays a vasculoprotective role and it has a beneficial effect by presumably forcing hepatocytes toward fatty acid oxidation and microsteatosis [31]. These findings are in line also with those of Solís-Muñoz et al. [38], who reported a similar trend in *ob/ob* mice intraperitoneally treated with melatonin at 10 mg/kg for 12 weeks [49].

Regarding the finding of increased in blood glucose concentration, we studied also the expression of RBP4, which is a blood circulating adipokine. We observed an increase in its expression in liver of *ob/ob* mice. We suggest that the enhanced expression could be linked to the rise in the glycemic value and may be a signal for the development of systemic insulin resistance. This suggestion is in agreement with other papers showing the increased levels of RBP4 obtained both in experimental animals and in humans [50]. This author suggested that, in mice, elevated levels of serum RBP4 led to impaired glucose uptake into muscle and induced glucose production by liver; whereas lowered serum RBP4 levels greatly enhanced insulin sensitivity. Thus, there is a possible link between obesity, glycemic value and insulin resistance. Further, in the current study we show that melatonin ameliorated this situation. On the basis of the these data, we suggest that melatonin regulates glucose production and determines its optimal production in both the liver and in the blood.

Herein, we characterized, by transmission electron microscopy analyses, the organization of mitochondria and RER and their physical distance from one another in *ob/ob* mice treated or not with melatonin. Melatonin reorganized RER and mitochondria mainly into round small mitochondria instead of megamitochondria, with a greater diameter, in the pericentral area of liver. Moreover, we demonstrated that melatonin expanded the strict juxtaposition between RER and mitochondria in the hepatocytes of *ob/ob* liver, by restoring the right distance as in control livers. This crucial ultrastructural finding may indicate the recovery of RER-mitochondria interplay, that was in line with reduced hepatic lipid accumulation and macrosteatosis. Indeed smooth or RER tethering to mitochondria is crucial for proper calcium flux from ER to mitochondria and for lipid deposition [51]. Increased MAM formation drives higher calcium accumulation in the mitochondria that in turn leads to impairment in mitochondrial oxidative capacity, increased ROS generation, cellular stress, impaired insulin action in the liver and abnormal glucose metabolism [52].

To confirm the involvement of ER and mitochondria in NAFLD, we studied also CNX, ATPSβ, GPR78 and CHOP by immunofluorescence. We observed an intense CNX positivity in *ob/ob* mice which is reduced with melatonin. This suggests an interaction of melatonin with CNX which determines calcium homeostasis. This data is in agreement with the finding of Macías et al. [53] who demonstrated that melatonin binds to calreticulin, another stress protein and its binding is very specific. In particular, calreticulin is a ubiquitous Ca^2+^ (calcium)-binding protein of the ER that may be regulated through intracellular signaling pathways involving Ca^2+^ binding. This protein, like CNX, is multifunctional and plays an important role in the modulation of a variety of cellular processes. These functions include chaperon activity, control of intracellular Ca^2+^ homeostasis and regulation of cell adhesiveness by interacting with the integrins at the cytoplasmatic site of the plasma membrane. Moreover, a recent paper reported that CNX is an integral ER membrane protein and molecular chaperone involved in the folding and quality control of membrane associated and secreted proteins, including many cell surface receptors regulated by tyrosine phosphorylation [54]. This protein crosses the ER membrane with a portion of the C-terminal domain residing in the cytoplasm where it may play a role in substrate folding. The C-terminal domain undergoes post-translational modifications including palmitoylation. Palmitoylation of the C-terminal domain mediates its association with the ribosome-translocon complex inducing the association of CNX with the mitochondria associated region of the ER. This post-translational modification indicates that the C-terminal domain of CNX plays a functional role in linking ER lumenal events with cytoplasmic signaling.

With regard to ATPSβ, we found a low expression in obese liver mitochondria. This is consistent with the data of Wang et al. [14] indicating that ATPSβ is a marker of progression of steatosis and diabetic complications in rodent models. In contrast, melatonin supplementation in *ob/ob* mice increased ATPSβ expression; this rise is associated with amelioration of mitochondria morphology and reduction of diameter. Moreover, this result is in line with recent study by Agil et al. [55] in Zucker diabetic rat liver, where melatonin mitigated steatosis and mitochondria alterations. Furthermore, it is known that mitochondria are important target of melatonin action [56] and that melatonin has a direct role in mitochondrial homeostasis reducing oxidative stress and enhancing also ATP production [57–59], these may explain how mitochondrial ultrastructural damages, CNX and ATPSβ expressions are restored after melatonin treatment.

Our data also show that GRP78 and CHOP expressions were high in liver of *ob/ob* mice and they were reduced after melatonin treatment. These findings were expected in list of the observations of other colleagues showing that these proteins are essential for proper protein folding and assembly [60]. It is accepted that the expression of GRP78 identifies the state of ER stress and CHOP could regulate several apoptotic signaling pathways leading to cellular death. Moreover, proapoptotic CHOP has been reported previously in *ob/ob* mouse liver by Arruda et al. [52]; but we demonstrate that these changes were hampered by melatonin. This latter finding may be due to the well established anti-apoptotic effect of melatonin related to the modulation of intrinsic mitochondrial pathway [61].

Another important finding of this study is that SIRT1 expression and protein level are very weak in the nuclei of *ob/ob* hepatocytes and increased after melatonin treatment. In our previous work [62], we showed that SIRT 1 expression was reduced in murine hepatocytes and in mice fed a high fructose diet. SIRT1 is an important cellular survival protein and it believed to promote healthy ageing [63,64]. SIRT1 regulates glucose and lipid metabolism through its deacetylase activity and via its direct and indirect involvement in insulin signaling. Moreover, a hepatocyte deficiency of SIRT1 induced activation of several systems responsible for causing damage and decreasing fatty acid oxidation [65]. Remarkably, liver-specific SIRT1 deficiency causes hepatic glucose overproduction, chronic hyperglycemia and increased oxidative stress. Importantly, SIRT1 controls the redox system and reduces oxidative stress [18].

We also studied β-catenin expression and observed that it was recovered in *ob/ob* mice after melatonin treatment. This is strongly correlated with reduced macrosteatosis confirming that alterations in its activity is associated with the development of NAFLD and other liver diseases, as reported also by Monga [66]. This is because β-catenin also regulates the expression of genes that control the metabolism of glucose and nutrients. There are many pathways in which β-catenin is involved, including Wnt signaling. β-catenin also interacts with transcription factors such as T-cell factor, forkhead box protein O and hypoxia inducible factor 1α to regulate the expression of target genes. Moreover, alterations in its activity may contribute to the pathogenesis of NAFLD [66].

**Fig 6:**
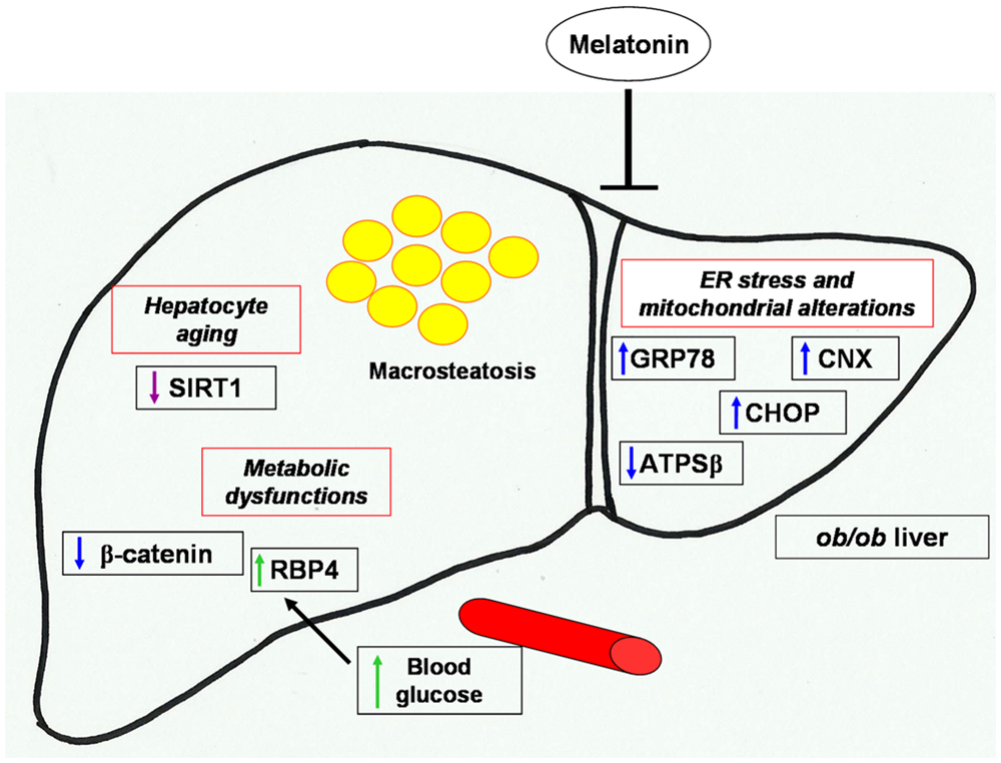
Melatonin protective effects against NAFLD liver alterations. Schematic representation of melatonin attenuation of hepatic macrosteatosis, endoplasmic reticulum stress, mitochondrial and metabolic alterations in NAFLD obese mice.

In conclusion, melatonin promotes several physiological pathways modulating hepatic and, in particular, liver mitochondrial and ER dysfunctions that are involved in obesity and NAFLD as a potential treatment for these diseases (Fig 6). This study may help to explain, together with the evidences of melatonin safety and efficacy, the benefits of melatonin against obesity and NAFLD. To understand the exact mechanism(s) underlying melatonin diet supplementation beneficial effects at cellular and molecular levels, broader studies using various (probably low) dosages of melatonin and varied durations of treatment at different stage of the obesity pathogenic process should be considered. Nevertheless, approaches that restore also the proper function of ER-mitochondria interface may be useful tools in the treatment of multiple metabolic diseases.
